# Abuse and revictimization in adulthood in multiple sclerosis: a cross-sectional study during pregnancy

**DOI:** 10.1007/s00415-022-11249-x

**Published:** 2022-07-03

**Authors:** Karine Eid, Øivind Torkildsen, Jan Aarseth, Elisabeth G. Celius, Marianna Cortese, Trygve Holmøy, Akash Kapali, Kjell-Morten Myhr, Cecilie F. Torkildsen, Stig Wergeland, Nils Erik Gilhus, Marte-Helene Bjørk

**Affiliations:** 1 Department of Neurology, Haukeland University Hospital, Jonas Lies vei 71, 5053 Bergen, Norway; 2 Department of Clinical Medicine, University of Bergen, Bergen, Norway; 3 Department of Neurology, Neuro-SysMed, Haukeland University Hospital, Bergen, Norway; 4 The Norwegian Multiple Sclerosis Registry and Biobank, Haukeland University Hospital, Bergen, Norway; 5 Department of Neurology, Oslo University Hospital, Oslo, Norway; 6 Institute of Clinical Medicine, University of Oslo, Oslo, Norway; 7 Department of Neurology, Akershus University Hospital, Lørenskog, Norway; 8 Department of Global Public Health and Primary Care, University of Bergen, Bergen, Norway; 9 Department of Obstetrics and Gynecology, Stavanger University Hospital, Stavanger, Norway

**Keywords:** Violence, Revictimization, The Norwegian Mother, Father, and Child Cohort study, MoBa, The Medical Birth Registry of Norway

## Abstract

**Background:**

Knowledge concerning exposure to abuse in adulthood and in pregnancy in people with multiple sclerosis (MS) is sparse.

**Objective:**

To determine the occurrence of adult abuse and abuse in relation to pregnancy in women with MS and their risk of revictimization (repeated abuse as adults after childhood abuse).

**Methods:**

This cross-sectional study comprised pregnant women from the Norwegian Mother, Father and Child Cohort study. Information on abuse was acquired through self-completed questionnaires. We used logistic regression to estimate adjusted odds ratios (aORs) with 95% confidence intervals (CIs).

**Results:**

We identified 106 women with MS at enrollment through linkage with national health registries. The reference group consisted of 77,278 women without MS. Twenty-seven women (26%) with MS reported any adult abuse compared to 15,491 women (20%) without MS, aOR 1.33 (0.85–2.09). Twenty-two (21%) women with MS reported systematic emotional abuse compared to 13% without MS, aOR 1.75 (1.08–2.83). Ten women (10%) with MS reported sexual abuse, compared to 6% without MS, aOR 1.72 (0.89–3.33). More women with MS reported rape as an adult, aOR 2.37 (1.02–5.49). Women with MS had higher risk of revictimization as adults, after childhood abuse, aOR 2.23 (1.22–4.10). The risk of abuse during pregnancy or 6 months preceding pregnancy was similar between the groups.

**Conclusions:**

Women with MS had increased occurrence of systematic emotional abuse, rape, and revictimization as adults, compared to women without MS.

**Supplementary Information:**

The online version contains supplementary material available at 10.1007/s00415-022-11249-x.

## Introduction

People with multiple sclerosis (MS) are more often exposed to abuse and neglect in childhood than the general population [1–4]. Mistreatment in childhood is a strong predictor of abuse later in life, known as revictimization [5]. It is not known whether abuse occurs more frequently in adulthood or during pregnancy for people with MS. However, people with physical impairment or activity limitations are at increased risk of experiencing any forms of sexual, physical, or emotional mistreatment [6, 7], including partner violence [8].

A US study found that 55% of people with advanced MS reported maltreatment by unpaid caregivers [9], most frequently emotional abuse. A focus group study found that people with advanced MS were reluctant to report being abused even though the caregiver admitted mistreatment [10]. No previous study has examined the occurrence of abuse in adulthood or the relationship to the abuser in general MS populations. Moreover, no study has examined the risk of experiencing abuse during pregnancy in women with MS.

Experiencing abuse has long-term consequences for mental and physical health [11]. Women who have previously experienced abuse may be more vulnerable for abuse during pregnancy [12]. Abuse during pregnancy is of particular concern due to the increased risk of adverse maternal and neonatal outcomes [13]. We have previously found that a history of physical or sexual abuse was a risk factor for perinatal depression in women with MS [14]. There is a need for increased attention to this issue to protect people with MS at risk and to support and provide trauma-informed care [15] for those in need.

Our aim was to investigate the occurrence of abuse in adulthood in pregnant women with MS and their risk of experiencing revictimization after childhood abuse. Further, we aimed to study their relationship to the abuser.

## Materials and methods

### Study design and data collection

We conducted a cross-sectional analysis based on questionnaire data from all women participating in the Norwegian Mother, Father, and Child Cohort Study (MoBa). MoBa is a nationwide, prospective cohort study, which included Norwegian-speaking pregnant women from all over Norway between 1999 and 2008 [16]. There were no exclusion criteria, and 41% of the invited women consented to participation. The MoBa cohort is linked to The Medical Birth Registry of Norway (MBRN), a nationwide medical registry containing information about all births in Norway. Registration of information in the MBRN is mandatory and performed by health personnel.

We acquired information on demographic and socioeconomic factors, medical history, and any experience of abuse from questionnaires self-administered during pregnancy weeks 17–20 and 30.

**Fig. 1:**
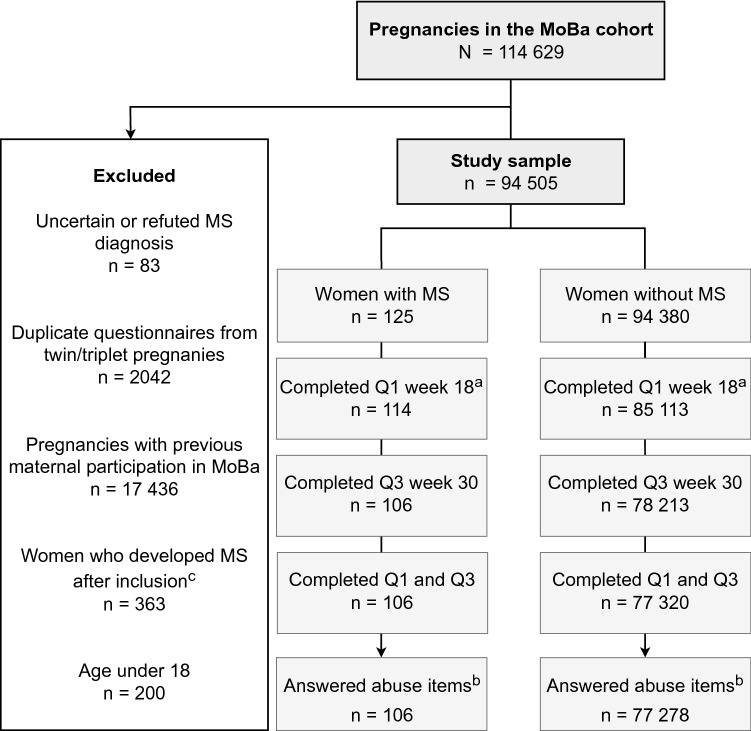
Flowchart of included and excluded study participants. *MoBa* The Norwegian Mother, Father and Child cohort study, *MS* multiple sclerosis, *Q* Questionnaire. ^a^Pregnancy week 17–20 (Q1). ^b^Women who completed the abuse questions in either week 17–20 (Q1) or week 30 (Q3) were included in our study. ^c^Women who developed MS after inclusion in MoBa until December 31, 2018 (date of data linkage) were excluded from the reference group

Our study is based on version 12 of the MoBa data files, covering 114,629 pregnancies. We included women who completed both the questionnaire in pregnancy week 18 and week 30, including the abuse items. To include only one observation per woman, we excluded duplicate questionnaires due to twin and triplet pregnancies and additional questionnaires from women with recurrent participations in MoBa (Fig. 1). We also excluded women who were under age 18 years at inclusion.

### MS diagnosis

To validate the self-reported MS diagnosis from the questionnaires, we cross-linked the MoBa cohort with the Norwegian Patient Registry (NPR) and the Norwegian Multiple Sclerosis Registry and Biobank (The MS Registry). We also included information from hospital records. After every consultation in specialist care, registration of all relevant diagnoses in NPR is mandatory for health practitioners. The MS diagnosis in NPR has a sensitivity of 97% and a positive predictive value of 0.92 [17]. We considered the MS diagnosis as validated if registered both in the NPR and in the MS registry. The MS registry had 69% national coverage at the time of data linkage [18]. If an MS diagnosis was registered only in NPR but not in the MS registry, we reviewed hospital records to validate the diagnosis using the 2017 diagnostic criteria for MS [19]. The linkage made it possible to identify women with MS who failed to report a history of MS at inclusion in MoBa (*n* = 4). We were also able to refute incorrect MS diagnoses from the NPR based on the information from the hospital records. NPR-identified MS cases not included in the MS registry and without access to the hospital records for validation were excluded (Fig. 1). This data linkage also identified women who developed MS after inclusion in MoBa up until December 31, 2018 (date of data linkage). These women were excluded from the main analyses but included in a sensitivity analysis.

### Abuse experience

#### Abuse categories

In pregnancy week 30, the women answered four questions concerning experiences of abuse (Questionnaire S1); emotional abuse—humiliation (“Has anyone over a long period of time systematically tried to subdue, degrade or humiliate you?”), emotional abuse—threat (“Has anyone threatened to hurt you or someone close to you?”), physical abuse (“Have you been subjected to physical abuse?”), and sexual abuse (“Have you been forced to do sexual actions?”). The question regarding humiliation was considered as systematic emotional abuse. The abuse questions in MoBa have been adapted from the NorVold Abuse Questionnaire showing good validity and reliability [20].

An experience of either emotional, sexual, or physical abuse as an adult was defined as responding “yes, as an adult > 18 years” to the respective categories.

Type and severity of sexual abuse in were assessed in the questionnaire in weeks 17–20; “Have you ever been pressured or forced to have sexual intercourse during this pregnancy, the last 6 months before pregnancy, or earlier?” The response options were “yes, pressured”, “yes, forced with violence” and “yes, raped.” We merged “forced with violence” and “rape” into one category of rape. This question did not distinguish between childhood and adulthood. We considered an experience of rape > 18 years of age if the woman also had reported sexual abuse as an adult in the questionnaire in week 30. Women who answered “no, never” were categorized as not having experienced rape.

#### Childhood abuse and revictimization

An experience of childhood abuse was defined as responding “yes, as a child < 18 years” to any of the abuse categories in the questionnaire in week 30. Women were defined as revictimized if they reported either emotional, sexual, or physical abuse both as a child (< 18 years) and as an adult (> 18 years).

#### Perpetrator

The questionnaire in week 30 included a question regarding the person responsible of abuse; “Who was responsible for this?”. The options were: “A stranger”, “Family or relative”, or “Another known person”.

#### Abuse during pregnancy or in the 6 months preceding pregnancy

The questionnaire in weeks 17–20 included two questions regarding whether the woman had experienced physical or sexual abuse during the current pregnancy or the last 6 months before pregnancy. These questions have been modified from the Abuse Assessment Screen, developed to detect abuse among pregnant women [21]. The women could also state in the week 30 questionnaire if the abuse had happened during the last 12 months. As the women were 7 months pregnant at this assessment, this comprised abuse during pregnancy and up to 5 months before pregnancy. Women who answered “yes” to either of these questions were defined as having experienced abuse during the current pregnancy or in the last 6 months before pregnancy.

### Covariables

MS-specific covariables were obtained from the MS registry and hospital records: Age at MS onset (defined as first clinical symptom), age at MS diagnosis, and subtype of MS (relapsing–remitting, primary progressive, or unspecified). Other covariables were acquired through the self-completed MoBa questionnaires or through linkage to the MBRN: age, smoking (ever/never), body mass index (BMI) prior to pregnancy (< 25/ ≥ 25 kg/m^2^), alcohol use ≥ 1 occasion per month during the first trimester or substance use (cannabis, amphetamine, ecstasy, cocaine, heroin) the last month before or during pregnancy. Adverse socioeconomic status in adulthood was defined as either having low household income (< 60% of the study population median income in the year of participation), being a non-cohabiting mother, or having low level of education (≤ 9 years of school). Low education level of the partner was defined as ≤ 9 years of school. Depression during pregnancy was measured by a validated short version of the Hopkins Symptom Checklist 25 [22], included in the same questionnaire as the abuse questions.

### Statistical analysis

The MS group was compared to a reference group of all women in MoBa without MS. We analyzed the risk for experiencing abuse by logistic regression with estimated odds ratios (ORs) and 95% confidence intervals (CIs). We considered age, history of smoking, overweight, and socioeconomic status (≥ 1 of the following: non-cohabiting mother, low level of education, low household income) as possible confounders and adjusted all models for these covariables. Low education of the woman’s partner was adjusted for in a secondary analysis when considering the person responsible of abuse, as this variable could potentially be a mediator for the association between MS and abuse. Depression was regarded as a collider and therefore not adjusted for [23]. Estimates with CIs not including 1 were considered statistically significant. Categorical variables were compared with the Pearson Chi-square test or Fisher exact test if any table cell count was expected to be < 5. Continuous variables were compared with t tests. We performed interaction analyses with logistic regression models by including interaction terms between the exposure (MS) and (1) low socioeconomic status and (2) childhood abuse on the outcome (adult abuse), adjusted for potential confounders. This was done to investigate whether women with MS were more susceptible to abuse as adults if they had low socioeconomic status or had experienced abuse in childhood. Statistical analyses were performed using IBM SPSS Statistics version 26 and Stata version 17 (StataCorp LLC).

#### Sensitivity analysis

As the questionnaires did not specify the exact period for the abuse experience, we lacked data on the timing of adult abuse with respect to the date of MS diagnosis. We therefore performed a sensitivity analysis comparing abuse risk in women with established MS to women who developed MS after inclusion in MoBa. The aim was to explore the direction of the associations. As women with future MS did not have the vulnerability of having a chronic condition [6, 8], higher rates of abuse in this group could signify that adult abuse predating the diagnosis could be risk or trigger factor for MS [24, 25], or associated with unknown confounders, rather than being a consequence of MS. In this analysis, we excluded women who had their first symptom of MS within 5 years after MoBa inclusion and could have been in a prodromal phase of MS [26].

## Results

We identified 106 eligible women with MS and 77,278 women without MS in the cohort at baseline. Women with MS tended to be more depressed, overweight, and with a history of smoking at study baseline, and they more often had a partner with low level of education (Table 1).

**Table 1:** Background characteristics of women with and without MS in MoBa

	Women with MS *n* = 106	Women without MS *n* = 77,278	*p* value
Age; mean (SD) [range]	31 (4) [21─42]	30 (5) [18─47]	0.02
Missing; *n* (%)	0 (0)	0 (0)	
Adverse socioeconomic status^a^; *n* (%)	9 (9)	8123 (11)	0.42
Missing; *n* (%)	1 (1)	15 (< 1)	
Low household income; *n* (%)	4 (4)	5492 (7)	
Low level of education; *n* (%)	< 3	1563 (2)	
Non-cohabiting mother; *n* (%)	4 (4)	1754 (2)	
Low level of education partner^b^; *n* (%)	10 (10)	3171 (4)	0.01
Missing; *n* (%)	8 (8)	7033 (9)	
Depression at study baseline^c^; *n* (%)	14 (13)	7162 (9)	0.15
Missing; *n* (%)	2 (2)	795 (1)	
Ever smoker; *n* (%)	57 (54)	39,357 (51)	0.61
Missing; *n* (%)	0 (0)	459 (1)	
BMI ≥ 25 kg/m^2^; *n* (%)	37 (35)	23,676 (31)	0.40
Missing; *n* (%)	1 (1)	1911 (3)	
Alcohol or substance use during pregnancy^d^; *n* (%)	4 (4)	2559 (3)	0.78
Missing; *n* (%)	0 (0)	0 (0)	
Age at MS diagnosis; mean (SD) [range]	26 (4) [14─36]	n/a	n/a
Missing; *n* (%)	7 (7)		
Age at MS onset^e^; mean (SD) [range]	24 (4) [14─36]	n/a	n/a
Missing; *n* (%)	7 (7)		
Type of MS		n/a	n/a
RRMS	94 (89)		
PPMS	< 3		
Uncertain	11 (10)		

*P* values are calculated from Pearson *χ*^2^ test or Fisher exact test for categorical variables, and *t* test of continuous variables

*MoBa* The Norwegian Mother, Father and Child cohort study, *MS* multiple sclerosis, *SD* standard deviation, *BMI* body mass index, *RRMS* relapsing remitting multiple sclerosis, *PPMS* primary progressive multiple sclerosis, *n/a* not applicable

^a^Adverse socioeconomic status is one of the following: non-cohabiting mother, low level of education ≤ 9 years of school, low household income (< 60% of the study population median in the enrollment year)

^b^ ≤ 9 years of school

^c^Depression was measured through validated short versions of the Hopkins Symptom Checklist-25 during pregnancy week 30

^d^Alcohol use ≥ 1 occasion per month during the first trimester or substance use (cannabis, amphetamine, ecstasy, cocaine, heroin) the last month before or during pregnancy

^e^MS onset defined as the first clinical symptom of MS

Twenty-seven women (26%) with MS reported any category of adult abuse compared to 15,491 women (20%) without MS, adjusted OR (aOR) 1.33 (0.85–2.09) (Table 2). The interaction term between MS and adverse socioeconomic status on the risk of any adult abuse yielded a *p* value of 0.041.

**Table 2:** Abuse as adults in women with and without MS

	Women with MS *n* = 106 Yes/no^a^; *n* (%)	Women without MS *n* = 77,278 Yes/no^a^; *n* (%)	OR (95% CI)	aOR^b^ (95% CI)
Any adult abuse	27 (26)/78 (74)	15,491 (20)/61,255 (80)	1.37 (0.88–2.12)	1.33 (0.85–2.09)
Emotional abuse	26 (25)/79 (75)	12,764 (17)/63,982 (83)	1.65 (1.06–2.57)	1.61 (1.03–2.53)
Systematic humiliation	22 (21)/83 (79)	9778 (13)/66,968 (87)	1.81 (1.13–2.91)	1.75 (1.08–2.83)
Threat	8 (8)/97 (92)	6065 (8)/70,681 (92)	0.96 (0.47–1.98)	0.93 (0.45–1.93)
Sexual abuse	10 (10)/95 (90)	4280 (6)/72,466 (94)	1.78 (0.93–3.42)	1.72 (0.89–3.33)
Rape^c^	6 (6)/86 (94)	1890 (3)/62,526 (97)	2.31 (1.01–5.29)	2.37 (1.02–5.49)
Physical abuse	3 (3)/102 (97)	4395 (6)/72,351 (94)	0.48 (0.15–1.52)	0.45 (0.14–1.42)
Abused during pregnancy or last 6 months before pregnancy^d^	9 (8)/97 (92)	5006 (6)/72,271 (94)	1.34 (0.68–2.65)	1.44 (0.72–2.86)
Revictimization: adult and childhood abuse	13 (16)/69 (84)	4964 (9)/52,055 (91)	1.98 (1.09–3.58)	2.23 (1.22–4.10)

Total N may differ for some of the abuse categories because of different response rates to the different abuse items and different definitions of «no abuse». Of the 106 women with MS, 1 woman answered the abuse questions in Q1 but not in Q3. Of the 77,278 women without MS, 532 women answered the Q1 abuse questions but not the Q3 abuse questions

*MS* multiple sclerosis, *OR* odds ratio, *CI* confidence interval

^a^«No» means “no adult abuse” for the respective type of adult abuse category (emotional, sexual, physical). For “rape”, «no» means no experience of sexual abuse. For “abused during pregnancy or last 6 months before pregnancy” «no» means either previous or no experience of abuse. For “Revictimization”, «no» means no exposure to neither childhood nor adult abuse

^b^Odds ratios are adjusted for age and adverse socioeconomic status

^c^Based on one question from the questionnaire in pregnancy weeks 17–20 (Q1) and combined with a report of sexual abuse as an adult in week 30 (Q3)

^d^Based on questions from the questionnaire in weeks 17–20 (Q1) (“during this pregnancy” or “last 6 months before pregnancy”) and the question in week 30 (Q3) (“have this occurred during the last 12 months”)

Twenty-two women (21%) with MS reported systematic emotional abuse in the form of humiliation compared to 9778 women (13%) without MS, aOR 1.75 (1.08–2.83). Ten women (10%) with MS reported sexual abuse, compared to 4280 women (6%) without MS, aOR 1.72 (0.89–3.33). Women with MS more often reported to have been raped as an adult (6% vs. 3%), aOR 2.37 (1.02–5.49). The risk of physical or emotional abuse in the form of threats was not increased. Nine women (8%) with MS reported that the abuse had happened during pregnancy or in the 6-month period before pregnancy, compared to 5006 (6%) women without MS, aOR 1.44 (0.72–2.86).

Twenty-two women (21%) with MS had experienced childhood abuse, compared to 14,164 women (19%) without MS, aOR 1.24 (0.77–2.0). Women with MS had a higher risk of experiencing revictimization as adults (abuse both in childhood and adulthood), aOR 2.23 (1.22–4.10) (Table 2). Interaction analysis indicated a synergistic effect between MS and a history of childhood abuse on the risk of experiencing adult abuse (*p* = 0.054).

For all categories of abuse, the most common abuser was “another known person” for both women with and without MS (Table S1). For emotional abuse, 7 women with MS (27%) reported a family member or relative as responsible compared to 2474 women (19%) without MS. Very few women (*n* < 3) with MS reported a stranger as the abuser. The risk of emotional abuse attenuated when adjusting for partner education in addition to the potential confounders, aOR 1.39 (0.86–2.26). The risk of sexual abuse was slightly increased, aOR 1.84 (0.95–3.58), after this additional adjustment. The risk of physical abuse remained unchanged.

### Sensitivity analysis

We found an increased risk of emotional abuse for women with MS when comparing them to women who developed MS in the future (≥ 5 years after study inclusion), aOR 2.79 (1.24–6.25) (Table S2). The aOR was 2.37 (0.76–7.46) for sexual abuse and 0.72 (0.15–3.55) for physical abuse.

## Discussion

Our study found an increased risk of emotional abuse as well as rape in adulthood in women with MS. For emotional abuse, the risk was highest for systematic humiliation. Furthermore, women with MS had a higher occurrence of revictimization compared to women without MS.

Our population-based study extends previous knowledge on abuse in women with MS. A previous cross-sectional study examined abuse by caregivers and found that this occurred in 55% of 206 people with MS who needed assistance or care from family or friends; this compared to 26% in our population. The previous study selected MS patients with advanced disease and had a response rate of only 17%. Thus, their prevalence estimates are not directly comparable.

We found an increased risk of revictimization in women with MS. The interaction analysis indicated that having experienced abuse in childhood may increase the risk of abuse in adulthood to a larger extent in women with MS than in women without MS. Childhood abuse is a known risk factor for abuse as adults in the general population [5]. Factors associated with an increased risk of revictimization are exposure to multiple forms of childhood abuse [5, 27] and feeling shame [28].

Women with MS most often reported “another known person” as responsible for all the types of adult abuse. When adjusting the estimates for low partner education, the risk of emotional abuse decreased. In contrast, the risk of sexual abuse increased. This may indicate an association between emotional abuse and a low education in the current partner, but not so for sexual abuse. Emotional abuse was the most common abuse category in our study, similar to the previous study on caregiver abuse [9]. Sexual abuse was the least reported type of abuse by the caregivers [9]. Caregivers of people with MS often experience high levels of stress [29]. Low level of education increased the risk for fatigue and mental health problems in caregivers of MS patients [30]. Caregiver mental health problems increased the risk for caregiver abuse in people with advanced MS [9]. Increased focus on information, support, and the healthcare needs of caregivers could therefore potentially reduce the abuse risk of women with MS.

We found an interactive effect between MS and an adverse socioeconomic status the risk of abuse, meaning that women with MS and adverse socioeconomic status were more susceptible to abuse compared to women without MS who had the same socioeconomic status. Other risk factors for abuse among adults with disabilities are depression, anxiety, and impaired cognition [7, 31–33]. Neurologists should be aware of these associations, as these symptoms occur with increased frequency in MS [34–36].

The risk of abuse in the months preceding or during pregnancy was not increased in women with MS compared to women without MS. However, as many as 8% of women with MS had experienced abuse in close relation to pregnancy. Abuse during pregnancy is of particular concern because of the increased risk of physical and mental pregnancy complications [13], including perinatal depression [14].

Strengths of our study include the use of a population-based dataset with a thorough validation of the MS diagnoses. We have detailed information regarding different categories of abuse, and we adjusted for relevant confounders. Our study has some limitations. We do not know the timing of the abuse in relation to the timing of the MS diagnosis. However, we found that women with established MS had higher risk of emotional abuse compared to women who got MS more than 5 years after our assessment. This suggests that women with MS may have experienced emotional abuse because of increased vulnerability due to a manifest disease [6–8]. Our study has a limited sample size, which resulted in few cases in some of the abuse subcategories. Women with MS in our study were young and had short disease duration, which may limit the generalizability to what people with MS experiences during the life and disease course. We had no information on MS severity. However, we studied pregnant women with MS, who constitute a physically healthy and less disabled part of the MS population with low Expanded Disability Status Scale scores [37–40]. Therefore, physical disability should not represent a major determinant for our findings. The MoBa cohort has a participation rate of 41%, which may result in lower generalizability. However, similar response rates are considered acceptable for large prospective studies [41]. Women with Norwegian ethnicity and high socioeconomic status are overrepresented in the MoBa cohort [42], which may influence the generalizability to the whole maternal population. Nonparticipation and the underrepresentation of women with adverse socioeconomic status may underestimate the abuse prevalence but should not affect the exposure-outcome associations [41–44].

In conclusion, we found increased risk of systematic emotional abuse, rape, and revictimization in adulthood in women with MS compared to women without MS. Women with adverse socioeconomic status had a particularly increased risk. Clinicians should be aware of these associations when treating women with MS, as abuse experiences have severe and long-term impact on physical and mental health.

## Supplementary Information

Below is the link to the electronic supplementary material.

Supplementary file1 (DOCX 26 KB)

## Data Availability

Enquiries regarding access to data from MoBa and the MBRN can be directed to the Norwegian Institute of Public Health. Data from the MS Registry are accessible for researchers by application [45].
