# Supplementary material for: Abuse and revictimization in adulthood in multiple sclerosis: a cross-sectional study during pregnancy
Source: J Neurol. 2022 Jul 3;269(11):5901–9. doi: 10.1007/s00415-022-11249-x (PMC9553842; doi:10.1007/s00415-022-11249-x)
Supplement: Supplementary file 1 — Supplementary file1 (DOCX 26 KB) [file 415_2022_11249_MOESM1_ESM.docx]

**Supplemental material for “Abuse and Revictimization in Adulthood in Multiple Sclerosis: A Cross-sectional Study During Pregnancy”. J Neurol**

Karine Eid, Øivind Torkildsen, Jan Aarseth, Elisabeth G. Celius, Marianna Cortese, Trygve Holmøy, Akash Kapali, Kjell-Morten Myhr, Cecilie F. Torkildsen, Stig Wergeland, Nils Erik Gilhus, Marte-Helene Bjørk

**Corresponding author:**

Karine Eid, MD

[karine.eid@uib.no](mailto:karine.eid@uib.no)

Department of Neurology, Haukeland University Hospital, Bergen, Norway

Department of Clinical Medicine, University of Bergen, Bergen, Norway

**Contents:**

**Questionnaire S1:** Abuse experiences from self-completed questionnaires in pregnancy weeks 17-20 and pregnancy week 30

**Table S1:** Perpetrator of adulthood abuse in women with and without MS

**Table S2:** Abuse in adulthood in women with MS and women who developed MS more than 5 years after assessment (future MS)

**Questionnaire S1: Abuse experiences from self-completed questionnaires**

**Q1: Pregnancy weeks 17─20**

| **Have you ever in your adult life been slapped, hit, kicked, or bothered in any way physically?** | During this pregnancy | Last 6 months before pregnancy | Earlier |
| --- | --- | --- | --- |
| Yes |  |  |  |
| No |  |  |  |
| Don’t remember |  |  |  |
|  | | | |
| **Have you ever been pressured or forced to have sexual intercourse?** | During this pregnancy | Last 6 months before pregnancy | Earlier |
| No, never |  |  |  |
| Yes, pressured |  |  |  |
| Yes, forced with violence |  |  |  |
| Yes, raped |  |  |  |

**Q3: Pregnancy week 30**

| **Have you ever experienced any of the following?** | No, never | Yes, as a child (under 18) | Yes, as an adult (over 18) | Who was responsible for this?  (A stranger/Family or relative/Another known person) | Has this occurred during the last 12 months?  (No/Yes) |
| --- | --- | --- | --- | --- | --- |
| Has anyone over a long period of time systematically tried to subdue, degrade, or humiliate you? |  |  |  |  |  |
| Has anyone threatened to hurt you or someone close to you? |  |  |  |  |  |
| Have you been subjected to physical abuse? |  |  |  |  |  |
| Have you been forced to do sexual actions? |  |  |  |  |  |

| **Table S1. Perpetrator of abuse in adulthood in women with and without MS** | | | |
| --- | --- | --- | --- |
| **Abuser** | **Women with MS**  **n (%)** | **Women without MS  n (%)** | **P-value^a^** |
| **Emotional abuse** | **26 (100)** | **12,764 (100)** | 0.52 |
| Stranger | < 3 | 906 (7) |  |
| Family/relative | 7 (27) | 2474 (19) |  |
| Another known person | 16 (62) | 9210 (72) |  |
| *Missing* | *< 3* | *174 (1)* |  |
|  |  |  |  |
| **Sexual abuse** | **10 (100)** | **4280 (100)** | 1.00 |
| Stranger | < 3 | 708 (17) |  |
| Family/relative | < 3 | 384 (9) |  |
| Another known person | 8 (80) | 3078 (72) |  |
| *Missing* | *< 3* | *110 (3)* |  |
|  |  |  |  |
| **Physical abuse** | **3 (100)** | **4395 (100)** | 1.00 |
| Stranger | 0 (0) | 689 (16) |  |
| Family/relative | 0 (0) | 771 (18) |  |
| Another known person | 3 (100) | 2816 (64) |  |
| *Missing* | *0 (0)* | *119 (3)* |  |

Based on the questionnaire in week 30. Cells with values less than 3 are censored
^a^P-values based on 2-sided Fisher exact test

| **Table S2. Abuse in adulthood in women with MS and women who developed MS more than 5 years after assessment (future MS)** | | | | |
| --- | --- | --- | --- | --- |
|  | **Women with established MS n = 106** | **Women with future MS**^a^ **n = 119** |  |  |
|  | **Yes/no abuse**^b^**; n (%)** | **Yes/no abuse**^b^**; n (%)** | **OR (95% CI)** | **aOR**^c^ **(95% CI)** |
| **Any adult abuse** | 27 (26) / 78 (74) | 22 (19) / 96 (81) | 1.51 (0.80–2.86) | 1.67 (0.82–3.40) |
| **Emotional abuse** | 26 (25) / 79 (75) | 15 (13) / 103 (87) | 2.26 (1.12–4.55) | 2.79 (1.24–6.25) |
| *Systematic humiliation* | 22 (21) / 83 (79) | 11 (9) / 107 (91) | 2.58 (1.18–5.62) | 3.08 (1.23–7.68) |
| *Threat* | 8 (8) / 97 (92) | 7 (6) / 111 (94) | 1.31 (0.48–3.74) | 2.26 (0.63–8.14) |
| **Sexual abuse** | 10 (10) / 95 (90) | 6 (5) / 112 (95) | 1.97 (0.69–5.61) | 2.37 (0.76–7.46) |
| Rape^d^ | 6 (6) / 86 (94) | 5 (5) / 93 (95) | 1.30 (0.38–4.41) | 1.44 (0.39–5.33) |
| **Physical abuse** | 3 (3) / 102 (97) | 6 (5) / 112 (95) | 0.55 (0.13–2.25) | 0.72 (0.15–3.55) |
| **Abused during pregnancy or last 6 months before pregnancy**^e^ | 9 (8) / 97 (91) | 8 (7) / 111 (93) | 1.29 (0.48–3.47) | 2.10 (0.65–6.81) |
| **Revictimization: Adult and childhood abuse** | 13 (16) / 69 (84) | 8 (10) / 76 (90) | 1.79 (0.70–4.58) | 2.60 (0.86–7.86) |

Abbreviations: MS = Multiple sclerosis; OR = Odds Ratio; CI = Confidence Interval

Total N may differ for some of the abuse categories because of different response rates to the different abuse items and different definitions of «no abuse».

^a^185 women in MoBa got their first symptom of MS > 5 years after study inclusion. 119 of them answered both the abuse items in pregnancy week 17–20 and week 30.

^b^ «No» means “no adult abuse” for the respective type of adult abuse category (emotional, sexual, physical). For “rape”, «no» means no experience of sexual abuse. For “abused during pregnancy or last 6 months before pregnancy” «no» means either previous or no experience of abuse. For “Revictimization”, «no» means no exposure to neither childhood nor adult abuse.

^c^ Odds ratios are adjusted for age and adverse socioeconomic status

^d^ Based on one question from the questionnaire in pregnancy weeks 17–20 and combined with a report of sexual abuse as an adult in week 30.

^e^ Based on questions from the questionnaire in weeks 17-20 (“during this pregnancy” or “last 6 months before pregnancy”) and the question in week 30 (“have this occurred during the last 12 months”).
